# Highly Efficient Hemostatic Cross-Linked Polyacrylate Polymer Dressings for Immediate Hemostasis

**DOI:** 10.3390/polym16060863

**Published:** 2024-03-21

**Authors:** Tong Ye, Zhiyuan Yang, Ruolin Hao, Jinnan Guo, Guifang Dou, Zhiyun Meng, Shuchen Liu, Ruolan Gu, Zhuona Wu, Yunbo Sun, Peng Han, Yiguang Jin, Hui Gan

**Affiliations:** Department of Pharmaceutical Sciences, Beijing Institute of Radiation Medicine, Beijing 100850, China; 17864291181@163.com (T.Y.); weiyinyinyueyue@163.com (Z.Y.); haoruolin99@163.com (R.H.); n17838391074@163.com (J.G.); douguifang@vip.163.com (G.D.); mengzhiyun@vip.163.com (Z.M.); liusc118@163.com (S.L.); gurl311@126.com (R.G.); wznphd@126.com (Z.W.); sunyunbo0919@126.com (Y.S.); 15901135949@163.com (P.H.)

**Keywords:** high-absorbent polyacrylate, polymer, hemostasis, vessel injury

## Abstract

A traumatic hemorrhage is fatal due to the great loss of blood in a short period of time; however, there are a few biomaterials that can stop the bleeding quickly due to the limited water absorption speed. Here, a highly absorbent polymer (HPA), polyacrylate, was prepared as it has the best structure–effectiveness relationship. Within a very short period of time (2 min), HPA continually absorbed water until it swelled up to its 600 times its weight; more importantly, the porous structure comprised the swollen dressing. This instantaneous swelling immediately led to rapid hemostasis in irregular wounds. We optimized the HPA preparation process to obtain a rapidly water-absorbent polymer (i.e., HPA-5). HPA-5 showed favorable adhesion and biocompatibility in vitro. A rat femoral arteriovenous complete shear model and a tail arteriovenous injury model were established. HPA exhibited excellent hemostatic capability with little blood loss and short hemostatic time compared with Celox^TM^ in both of the models. The hemostatic mechanisms of HPA consist of fast clotting by aggregating blood cells, activating platelets, and accelerating the coagulation pathway via water absorption and electrostatic interaction. HPA is a promising highly water-absorbent hemostatic dressing for rapid and extensive blood clotting after vessel injury.

## 1. Introduction

Hemorrhaging is the leading cause of human death in war, natural disasters, and traffic accidents, as well as during orthopedic, cardiovascular, and hepatic procedures [1,2,3]. Among these cases, the ratio of vessel trauma is relatively high, and over 30% of blood loss after trauma is life-threatening [4,5]. Thus, hemostasis is important in life-saving emergency medical treatments. Around one-third of mortalities are caused by excessive blood loss during severe physical trauma [6]. Using rapid hemostatic materials in the early stage of trauma bleeding can significantly reduce the amount of blood loss in the organism, save pre-hospital treatment time, and improve the survival rate of the injured. For bleeding extremities, easy-to-use gauze and bandages are preferred [7]. However, hemostatic devices such as gauze and bandages are not that effective when applying pressure. A tourniquet is the second option, but this can only be used for a short time to avoid ischemic necrosis of the distal limb [8,9,10]. Accordingly, there is a need to develop rapid hemostatic materials to address bleeding problems during vascular injuries [11].

Hemostatic materials can achieve hemostasis in various ways [12,13,14,15]. Ordinary hemostatic gauze can achieve hemostasis through physical compression [16]. Hemostatic sponges achieve hemostasis by absorbing local blood and expanding their volume to exert pressure on local tissues, such as polyurethane (PU) sponges [17,18,19]. Porous hemostatic materials achieve hemostasis by absorbing blood and concentrating coagulation factors (e.g., zeolite-based hemostatic powders) [20]. Polysaccharide-based hemostatic materials achieve hemostasis via the adsorption and activation of blood cells through their electrical charge (e.g., chitosan) [21,22,23]. Natural inorganic-based hemostatic materials can achieve hemostasis by activating the coagulation pathway (e.g., kaolin) [24,25]. In addition, dual hemostasis can be achieved by loading hemostatic drugs through hemostatic materials, and gelatin sponges loaded with thrombin can achieve excellent hemostasis when used in liver bleeding models [26].

Polyacrylate is widely used because of its straight chain structure and network structure. Straight-chain structures have been studied as pH-responsive nanomaterials because its conductive properties act as electrolytes to extend battery life [27,28,29]. Due to its good performance regarding water absorption, polyacrylates, with a network structure, can absorb pollutants such as metal ions and organic matter after environmental treatment [30,31]. In particular, sodium polyacrylate with a high specific surface area and good water absorption is expected to be an ideal hemostatic material. However, research on the highly absorbent polymer (HPA), polyacrylate, as a single hemostatic active agent, is rarely reported. Additionally, composites of HPA, with other components such as chitosan or collagen, have been investigated for hemostatic applications; all include HPA as an excipient to enhance the mechanical properties of the main components by exploiting its thickening and adhesive properties [32,33]. Nonetheless, the hemostatic mechanism of HPA is unclear. Thus, research into the use of HPA as a polymer hemostatic material is warranted.

Here, for the first time, we elucidated the activity and mechanism of hemostasis using HPA alone. A three-dimensional reticulated particle HPA, with high water absorption, was prepared, and characterized using Fourier transform infrared spectroscopy (FT-IR). The Zeta potential for its physicochemical properties, and their hemostatic effect, was verified by the in vitro clotting time, a rat femoral arteriovenous complete shear model, and a tail arteriovenous injury model. Furthermore, we conducted a comprehensive evaluation on the safety of this HPA polymer. Next, we investigated the relationship between the physical and chemical properties of HPA and the hemostatic mechanism by using thromboelastography, blood cell aggregation activation, and FXII factor interaction. This work demonstrates that HPA is a safe and promising hemostatic material for vascular injuries.

## 2. Materials and Methods

### 2.1. Materials

Acrylic acid, *N*′*N*-methylenebisacrylamide (MBA), and potassium persulfate (KPS) were purchased from Macklin Biochemical Technology Co., Ltd. (Shanghai, China). Paraformaldehyde, sodium hydroxide, and anhydrous ethanol were purchased from Sinopharm Co., Ltd. (Shanghai, China). Phosphate-buffered, saline-simulating, body fluid (PBS), Dulbecco’s modified Eagle’s medium (DMEM), and fetal bovine serum, were provided by Gibco Co., Ltd. (Encinitas, CA, USA). Lactate dehydrogenase (LDH) was purchased from the Nanjing Jiancheng Institute of Biology Co., Ltd. (Nanjing, China). MTT and calcein AM·PI were purchased from SolarBio Co., Ltd. (Beijing, China). A rat coagulation factor XII (F12) ELISA kit was purchased from Abbexa Co., Ltd. (Cambridge, UK). The thromboelastography kit was purchased from Shenzhen Udi Technology Co., Ltd. (Shenzhen, China). Deionized water was prepared using a Milli-Q purification system (Burlington, MA, USA). All animal procedures were performed in accordance with the ARRIVE guidelines, and they were approved and reviewed by the Beijing Institute of Radiation Medicine (Beijing, China, IACUC-DWZX-2020-503); of which, the exact date of ethical permission was 30 August 2021.

### 2.2. HPA Synthesis

An acrylic acid solution of 48 mL was mixed with 62 mL of 30% sodium hydroxide solution, then, an initiator and cross-linking agent were added, at different ratios, and finally, deionized water was added to a constant volume of 200 mL and named MBA_n_/KPS_m_ HPA (n = 1, 2, 3, which means 0.03%, 0.06%, and 0.12%; m = a, b, c, which means 0.05%, 0.10%, and 0.25%.), as shown in Table 1. After mixing, it was stirred at 65 °C and 100 rpm until the gel product was fully formed. After cooling to room temperature, the gel was washed three times with 95% ethanol. Then, the gels were cut into approximately 2 × 2 × 0.5 cm^3^ blocks, and immersed in anhydrous ethanol for 12 h. Next, the polymer was dried and pulverized at 70 °C using a 40–100 mesh screen.

### 2.3. Structural Characterization

FT-IR spectral data of HPAs were collected using a Nicolet iS50 infrared spectrometer (Waltham, MA, USA) between 4000 and 500 cm^−1^, at a resolution of 4 cm^−1^, and 32 scans were accumulated. HPA morphology was observed using SEM (Tescan Mira4+ Spectrum xplore 30 EDS, Pleasanton, CA, USA). The swollen HPA-5 was transferred into liquid nitrogen for rapid freezing, the sample was excised, freeze-dried, and gold sprayed for observation. The Zeta potential of HPAs was ascertained using laser Doppler electrophoresis (Zetasizer Nano ZS90, Malvern Instruments Ltd., Malvern, UK). The thermal stability of HPAs was measured using a Discovery TGA (TG-DTA7300, Tokyo, Japan) between 30 and 800 °C with a resolution of 10 °C min^−1^ under an N_2_ atmosphere.

### 2.4. Water/Saline Absorption Ratio and Swelling Ratio Measurement

The water absorption of dried HPAs was studied in accordance with a general gravimetric method. Dried polymers (W_d_) were soaked in water or saline at 37 °C. Then, swollen gels were extracted at a specified interval, carefully wiped with a bibulous paper, and weighed (W_w_). Similarly, the swelling capacity of the dried HPA was analyzed based on the gravimetric method. Dried polymers (W_d_) were soaked in water or saline at 37 °C. Then, the swollen gels in the mesh screen were taken out after 30 min, carefully wiped with a bibulous paper, and weighed (W_w_). The absorption ratio was calculated as follows:

Water absorption (%)/swelling rate (%) = (W_w_ − W_d_)/W_d_ × 100%, (1)

### 2.5. In Vitro Hemostatic Assay of HPA

The in vitro coagulation time of whole blood (CBT) aimed to evaluate the effect of blood clotting on hemostatic materials. HPAs, with theoretically better hemostatic activity, were screened using the relationship between physicochemical properties and hemostasis. Precisely weighed 5, 10, 20, and 30 mg samples (HPA and Celox^TM^) were placed in 5 mL centrifuge tubes; Celox^TM^ was used as a positive control and blank blood was used as a negative control. Anticoagulated whole blood (1 mL) and CaCl_2_ (0.2 M, 30 μL) was added to each tube to initiate coagulation. The samples were placed in a water bath at 37 °C and tilted at 15 s intervals. The time for the blood to completely clot was recorded.

For the in vitro blood clotting index (BCI) test, samples (HPA and Celox^TM^) were accurately weighed at 5, 10, 20, and 30 mg, and placed in 10 mL centrifuge tubes. Anticoagulated whole blood (1 mL) and CaCl_2_ (0.2 M, 20 μL) were added to initiate coagulation at 37 °C for 5 min. The blank group included anticoagulated whole blood, and Celox^TM^ was used as a positive control. Deionized water (5 mL) was added and incubated at 37 °C for 10 min, causing the uncoagulated blood cells to swell and rupture. The absorbance of the sample supernatant was measured at 545 nm and marked as a, and that of the blank was b [34,35]. The BCI was calculated as follows:

BCI (%) = a/b × 100%(2)

### 2.6. Cytotoxicity Assay

HPAs with better hemostatic activity were screened using in vitro coagulation performance assays for the following experiments. A Methyl thiazolyl tetrazolium colorimetric assay (MTT) and a Calcein-AM/PI cell double staining kit were used to evaluate the HPAs’ cytocompatibility. Mouse fibroblasts (L929) were inoculated into 96-well plates at a density of 1500 cells/well. A HPAs infusion diluent (100 μL) was co-cultured with L929 for 24, 48, and 72 h. The blank group comprised only a DMEM complete culture medium, and the positive control comprised a DMEM culture medium with phenol (100 μL, 0.2%). The liquid in each well was discarded, and an MTT (50 μL, 1 mg/mL) solution was added to each well and incubated for 2 h. Then, isopropanol (100 μL) was added to each well and shaken for 10 min until fully dissolved. To calculate cell viability, the absorbance of each well was measured at 570 nm and 650 nm (calibration wavelength) [33]. Each sample had six repeat wells. The relative growth rate (RGR) was calculated as follows:

RGR (%) = OD_sample_ [OD_570_ − OD_650_]/OD_blank_ [OD_570_ − OD_650_] × 100%, (3)

L929 was inoculated into 6-well plates at a density of 10,000 cells per well. A HPAs infusion diluent (1 mL) was cultured with cells for 24, 48, and 72 h. The blank included L929 cells cultured in a DMEM-complete medium. Calcein-AM and PI were used for the simultaneous double staining of living and dead cells. The levels of living and dead cells were analyzed using an inverted fluorescence microscope. Each sample had three repeat wells.

### 2.7. Hemolysis Assay

Fresh anticoagulated rat whole blood (5 mL) was diluted with saline (5 mL) and used within 2 h. HPAs (5, 10, 20, and 30 mg), 200-μL diluted anticoagulated whole blood, and 5 mL saline were added to each tube in all sample groups, and they were incubated in a shaker at 37 °C for 1 h. Deionized water and saline were used as positive and negative controls, respectively. Thereafter, the harvested blood cell suspension was centrifuged at 3000 rpm for 5 min, and 3 mL of the supernatant were transferred to a cuvette. The OD value at 545 nm was measured using an ultraviolet spectrophotometer [36]. The hemolysis rate was calculated as follows:

Hemolysis rate (%) = (OD_samples_ − OD_negative_)/(OD_positive_ − OD_negative_), (4)

### 2.8. Acute Toxicity Assay

HPA with optimal hemocompatibility and cytotoxicity were screened using hemolysis and cytotoxicity assays for the following experiments. Ten healthy Kunming mice were randomly divided into two groups. In the experimental group, mice were intraperitoneally injected with a 50 mL/kg HPA infusion, whereas in the control group, they were injected with 0.9% NaCl. The immediate responses after administration were recorded, together with daily manifestations of systemic toxicity at 4 h and after 1–7 days. Mouse weight was recorded daily throughout the entire experiment.

### 2.9. Adhesion Property Test

Lap-shear strength tests were used to assess the adhesive properties of HPA on glass, Al_2_O_3_, plank, and pig skin. The experiments were designed to investigate the adhesion of HPA to different materials. In the experiments focusing on the adhesion properties of HPA on glass, Al_2_O_3_, and planks, HPA was in a gel state before being dried. However, in the adhesion experiments on biological samples (e.g., pig skin), dried HPA particles were used. Two substrate materials (rectangular sections with dimensions of 75 × 15 mm^2^) adhered to HPA with an overlapping area of 15 × 15 mm^2^. The sandwich sample was pressed using a 200 g weight for 20 min at room temperature. Next, the adhesion strength was assessed using a tensile strength test machine (Dongguan Huitai Machine Co., Ltd., Dongguan, China) at 10 mm/min [37,38,39]. The adhesion strength was calculated as follows:

τ = *F*/*s*, (5)

where τ denotes the adhesion strength (MPa), *F* is the tensile strength (N), and *s* is the overlapping area.

### 2.10. In Vivo Hemostatic Assay

Male Sprague–Dawley (SD) rats (weight 220 ± 20 g) were randomly divided into different groups. All animals were handled in accordance with the guidelines from the National Institutes of Health. To evaluate the potential of HPA as hemostatic material, we used it to treat a rat tail-amputation hemostatic model and a femoral arteriovenous complete incision model.

### 2.11. Tail Arteriovenous Injury

SD rats were anesthetized via inhalation and fixed on surgical plates. Their tail was completely cut off at half of the tail length. The wound remained naturally bleeding for 15 s, before applying 0.5 g of HPA to the incision site without compression. Celox^TM^ and a blank were used as controls. The hemostasis time was recorded, and blood loss in each group was weighted [36].

### 2.12. Femoral Arteriovenous Complete Shear

SD rats were anesthetized via inhalation and fixed on surgical plates. The hair around the left thigh was removed with operating scissors, and the surgical area was disinfected with 75% alcohol. Then, the skin and soft tissues were cut using ophthalmic scissors to expose the femoral artery, vein, and nerve. The uncontrolled femoral vascular hemorrhage model was created by completely severing the rat’s femoral artery and vein with ophthalmic scissors. After the vessels freely bled for 10 s, 0.5 g of HPA was applied over the injury site at a specific pressure until the bleeding stopped. Controls were treated only with pressure from Celox^TM^ and a standard gauze, respectively. After the bleeding stopped, clotting time, blood weight, and clotted materials in the groin were measured. After euthanizing the rats, a histological analysis of the wound site with the femoral arteriovenous vessels in rats was taken for H&E staining.

### 2.13. Preparation of Platelet-Rich Plasma (PRP) and Platelet-Poor Plasma (PPP)

Anticoagulated rat whole blood was centrifuged at 800 rpm for 10 min to obtain PRP, and PPP was collected from the blood supernatant after centrifuging at 3000 rpm for 10 min. The platelets in PRP were counted using a hemocytometer, which was adjusted via PPP to 1 × 10^8^/mL.

### 2.14. Morphology of Adsorbed Platelets and Erythrocytes

The HPA was immersed in a 5% platelet and erythrocyte suspension, respectively, and incubated at 37 °C for 1 h. The HPA was then washed three times with PBS to remove nonadherent platelets and red blood cells (RBCs). Then, the aggregated platelets and RBC were fixed with 2.5% glutaraldehyde for 4 h. Subsequently, samples were dehydrated with 20%, 50%, 80%, and 100% ethanol in a 10 min gradient. After samples were frozen-dried, they were gold-sprayed for SEM [13].

### 2.15. Adherent Platelet Quantitative Analysis

A mixture of 2 mL adjusted PRP and 10 mg of HPA was incubated at 37 °C for 30 min, followed by centrifugation at 500 rpm for 10 min. The precipitate of PRP with HPA was washed with PBS to remove nonadherent platelets. Then, 2% Triton X-100 was added to the plate to lyse platelets at 37 °C for 1 h. Next, the supernatant was collected via centrifugation at 10,000× *g* for 15 min, and the lactate dehydrogenase activity of platelets was determined using the LDH Kit (Nanjing Jiancheng Institute of Biology Co., Ltd., Nanjing, China). The number of adhered platelets was counted using the calibration curve of the lactate dehydrogenase. PRP was used as a control. All experiments were performed in triplicate.

### 2.16. Platelet Activation Was Measured Using Flow Cytometry

Platelet activation was assessed using a fluorescence activated cell sorting instrument (Guava easyCyte, Millipore Corporation, Hayward, CA, USA). First, 5 mg of HPA and 200 μL of PRP were incubated at 37 °C for 30 min. Then, the mixture was washed with PBS, centrifuged at 1200× *g* for 10 min, and the supernatant was discarded. Afterwards, 200 μL of PBS was added to the precipitate to form a platelet suspension. Platelets were used as a blank group. Thereafter, APC-CD62P and PE-CD61 were added to the platelet suspension and incubated for 20 min at 4 °C [40]. The marked platelets were washed three times with a cell staining buffer and tested.

### 2.17. Adherent Erythrocyte Quantitative Analysis

Fresh RBCs were prepared after the centrifugation of rat blood at 800 rpm for 10 min. The RBCs were washed three times with PBS and diluted in a 5% (*v*/*v*) erythrocyte suspension in PBS, which was placed under an UV spectrophotometer and scanned sequentially from 200 nm to 900 nm to obtain a maximum wavelength of 540 nm. In a 10-mL centrifuge tube, 25 mg of HPA and a 5 mL erythrocyte suspension (5% *v*/*v*) were incubated for 30 min at 37 °C. Afterwards, 3 mL of the suspension was lysed by adding deionized water at a 1:4 ratio (*w*/*v*), and the absorbance was measured at 540 nm. No material was added to the control RBC suspension. The number of RBCs that adhered to the surface of the HPA was then calculated. All experiments were repeated in triplicate.

### 2.18. Thromboelastogram (TEG) Analysis

This was performed using a UD-T5000 (UD-Bio, Shenzhen, China). HPA was mixed with anticoagulated whole blood at 5 mg/mL. Then, 1 mL of the mixture was added to reagent 1 in order to obtain the test blood, which was incubated for 1 min to activate blood coagulation. Test cups were placed on a thromboelasticity apparatus, each with 20 μL of reagent 2 (CaCl_2_); then, 340-μL of test blood was added for detection. Anticoagulated whole blood was used as a blank. All experiments were repeated in triplicate.

### 2.19. Activated Partial Thromboplastin Time (APTT) and Prothrombin Time (PT) Analysis

HPA was investigated for the activation of endogenous or exogenous coagulation pathways by testing the APTT and PT. A total of 10 mg of HPA was mixed with 5 mL of PPP and incubated at 37 °C for 30 min. The upper serum layer was placed in a RAC-030 automatic coagulation analyzer to react with APTT and PT reagents, respectively.

### 2.20. Determination of Coagulation FXII Factors

To explore the interaction between the negative charge on the HPA surface and coagulation FXII factors, heparin sodium anticoagulated whole blood was centrifuged at 1000× *g* for 15 min, and plasma was prepared. The plasma served as a blank. In the experimental group, HPA was added to plasma and incubated for 30 min. Then, the supernatant was collected via centrifugation at 1000× *g* for 15 min. Every sample in both the blank and experimental groups was diluted 20 times, and the amount of the FXII factor in plasma was determined in accordance with the Rat Coagulation Factor XII ELISA Kit instructions. Changes in the FXII factor in plasma, before and after the addition of HPA, were calculated, based on the established standard curve of the FXII concentration–OD value. The amount of FXIIa, tumor necrosis factor-α (TNF-α), and interleukin-6 (IL-6) factors in plasma, using a HPA and blank plasma, was quantified using similar methods.

### 2.21. Statistical Analysis

All statistics were analyzed using GraphPad Prism (GraphPad Software 8.0.2, Santiago, CA, USA) and Origin software (OriginLab 8.0, Northampton, MA, USA). The quantitative data were shown using the mean ± standard deviation (SD) of at least three tests. One-way ANOVA was performed to assess significant differences, and a value of *p* < 0.05 was considered to be statistically significant.

## 3. Results and Discussion

### 3.1. Characteristics of HPA

To prepare a HPA polymer with a three-dimensional network structure, we chose a neutralization level of 70% and used NaOH to neutralize the acrylic acid. The dosages of MBA as a crosslinker and KPS as an initiator were critical for the preparation of HPA. Therefore, nine HPAs with different composition ratios were synthesized.

**Figure 1:**
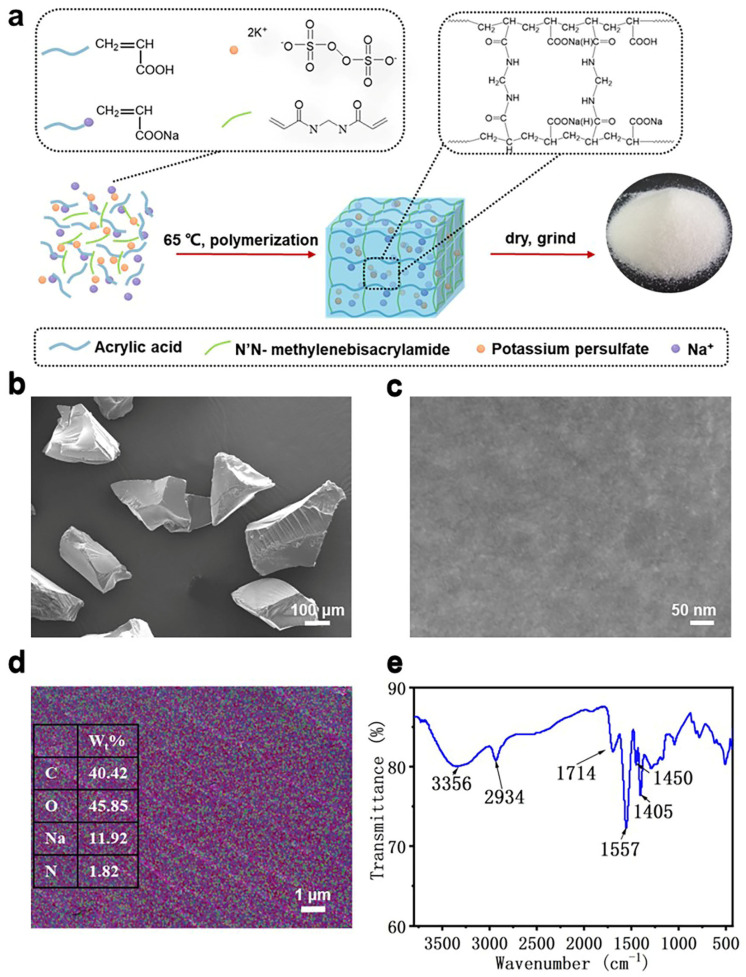
Synthesis and structural characteristics of HPA. (**a**) Preparation of HPA particles. (**b**,**c**) Scanning electron microscopy of HPAs. (**d**) Energy dispersive spectrum of HPA-5: red represents the C element, green represents the N element, purple represents the Na element, and blue represents the O element. (**e**) Fourier transform infrared spectroscopy characteristic bands of HPA.

The SEM results showed the surface morphology and porous structure of HPA. Its particle size was 100–200 μm (Figure 1b). The porous structure of HPA particles cannot be observed at a 100 k× magnification (Figure 1c). With energy dispersive spectrum (EDS) mapping, we could observe a uniform distribution of C/N/O/Na elementals on the HPA’s surface (Figure 1d and Figure S1). The cross-linking degree of the HPA surface can be demonstrated laterally using the N/C element ratio on the HPA surface, which, in turn, demonstrates its swelling capacity. When an equal amount of initiator KPS was used, the N/C ratio became larger with the increase in crosslinker MBA (Table S1). And when an equal amount of MBA was used, KPS_b_ had the biggest N/C ratio, which indicated that an adequate amount of initiator can determine the amount of N introduced. It was presumed that a low dose of KPS triggered a small amount of free radical production, which reduced the cross-linking degree of HPA. The excess acrylic acid radicals generated increased KPS content, which underwent self-polymerization, thus affecting the cross-linking of MBA with acrylic acid. Thus, KPS_b_ is the better initiator dose for the synthesis of network HPAs.

FTIR clarified the characteristic groups of HPA, as shown in Figure 1e. The characteristic spectral band of 3356 cm^−1^ corresponded with the -OH stretching vibration band. The feature spectral band of the -CH_2_ asymmetric stretching vibration was found at 2934 cm^−1^. In particular, a sharp and strong band near 1700 cm^−1^ corresponded with the characteristic band of carbonyl (C=O); 1557 cm^−1^ was the characteristic spectral band of the seco-amide-conjugate, and 1450 and 1404 cm^−1^ were those of the -CO bending vibration [41,42,43,44]. Based on SEM and FTIR results, we determined the successful synthesis of a polyacrylate polymer.

### 3.2. Water and Saline Absorption and Swelling Abilities of HPA

**Figure 2:**
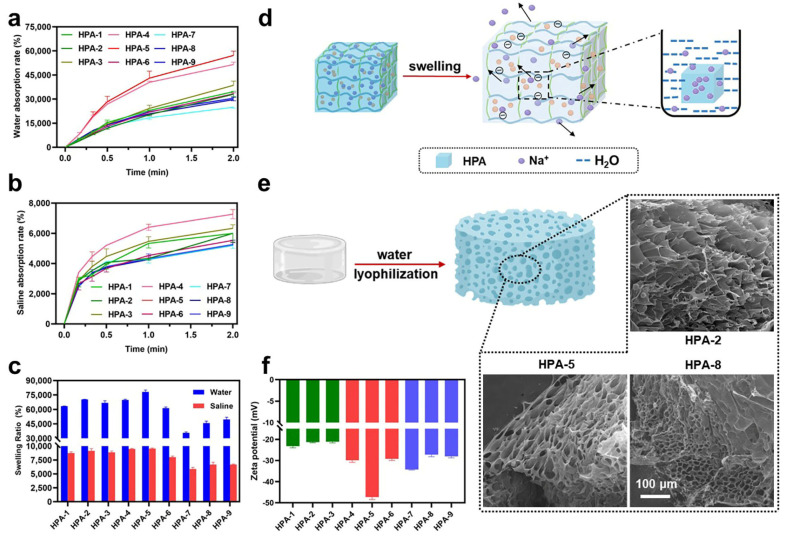
Physicochemical properties of HPA. (**a**) Water absorption ratio of MBA_n_/KPS_m_ (HPA1–9) group, *n* = 3. (**b**) Saline absorption ratio of the MBA_n_/KPS_m_ (HPA1–9) group, *n* = 3. (**c**) Swelling ratio of the MBA_n_/KPS_m_ HPA group, *n* = 3. (**d**) HPA’s water absorption mechanism. (**e**) Scanning electron microscopy of the freeze-dried MBA_n_/KPS_b_ HPA after complete swelling in water. (**f**) The Zeta potential of HPA1–9 group, *n* = 3.

Water absorption and swelling are important factors for hemostatic materials [44], as it allows them to expand during the clotting cascade reaction after quickly absorbing water from the blood and concentrating the clotting factors. Compared with other HPA ratios, HPA-4 (51,530 ± 5210% in deionized water and 7270 ± 310% in saline) and HPA-5 (57,200 ± 2650% in deionized water and 7270 ± 310% in saline) exhibited excellent aspiration rates within 2 min (Figure 2a,b). A higher absorption capacity was found in pure water. The presence of salts produces a homo-ionic effect, which significantly affects the absorptive capacity of the resin.

When in contact with HPA, water molecules penetrate HPA through capillary action and diffusion. The Na^+^ on the HPA chain is ionized in water, and the Coulomb repulsion it generates holds open the three-dimensional structure and draws water into the resin, with the help of the osmotic pressure generated by the Na^+^ inside and outside the ionized HPA (Figure 2d). In short, HPA interacted with water molecules and stretched from a tight state to a three-dimensional mesh structure, along with the absorption of water molecules. Therefore, it is able to absorb a quantity of water that is many times greater than its original weight.

The higher the swelling rate, the better the ability to concentrate clotting factors [45]. The swelling rate (SR) of particles in deionized water and saline is shown in Figure 2c. When HPA was immersed in deionized water or saline for 20 min, the dissolution rate reached a stable level. For the nine MBA_n_/KPS_m_ HPAs, the SR_water_ was >61,330 ± 1170 and SR_saline_ was >8000 ± 200%, which indicated the successful synthesis of a polyacrylate polymer with high water absorption. The dissolution rates of HPA1–6 were higher than those of HPA7–9. In particular, HPA-5 exhibited the maximum SR (9530 ± 120% in saline, 78,130 ± 2050% in deionized water). In this study, the prepared HPA had a higher dissolution rate, relative to HPA composites [13,33,36]; this indicated that it promoted the deposition of more platelets and erythrocytes on the surface of the material, which is more conducive to hemostasis. Then, HPAs were freeze-dried after being thoroughly soaked in water, and the results of SEM showed that HPA had a dense porous structure after water absorption (Figure 2e). The density of the pores of the MBA_n_/KPS_b_ HPA became more dense as the crosslinker MBA increased, indicating that the higher the MBA dosage, the higher the resin cross-linking density, and as such, the HPA could not sufficiently absorb water and swell. In particular, the pore size of HPA-2 was about 68 μm, the pore size of HPA-5 was about 45 μm, and the pore size of HPA-8 was about 22 μm. The results indicate that the crosslinker is unfavorable for HPA swelling when it is in excess. Thus, MBA_3_/KPS_m_ (HPA7–9) exhibited lower absorption and SRs, which may have been caused by the excessive crosslinking of the pre-existing monomer. MBA_1_ and MBA_2_ are thus the more suitable crosslinking agent dosages.

Negatively charged particles on the surface activated coagulation factors XII to XIIa, thus triggering the endogenous coagulation pathway [46]. In Figure 2f, the Zeta potential of HPA1–6 showed a large negative charge on the surface in the water system, which is related to the swelling of HPA. This occurs when the Na^+^ on the HPA allows contact between the polymer ionic chain and water, exposing the carboxyl groups on the polymer and making the HPA surface negatively charged. Mutual negative electrical repulsion between carboxyl groups leads to the rapid stretching of the polymer web bundles from an intertwined state. Water absorption using HPA frees more Na^+^ within the polymer, exposing more carboxyl groups, and thus absorbing more water. Thus, the strength of the negative charge in HPA also indicates swelling capacity. The charge of MBA_1_/KPS_m_ (HPA1–3) was smaller than that of MBA_2_/KPS_m_ (HPA4–6), as shown in Figure 2e, probably because of the low level of the crosslinker in the synthetic stage, resulting in the presence of fewer network polymers in the reaction. HPA-5 (−47.30 mV) had the strongest negative charge and the best swelling ratio. This is due to the stronger osmotic pressure generated by Na^+^ inside and outside the HPA.

The thermal stability of HPA is shown in Figure S2. HPA4–6 lost about 8% of its weight < 100 °C via free water evaporation. From 340 °C to 430 °C, the weight loss was about 12%, presumably due to the decomposition of some linear oligomers in the sample. Thus, HPA has high temperature resistance and is suitable for stable storage as a biomedical material [29]. Additionally, HPA4–6 had the best water absorption, swelling ratio, negative charge, and thermal stability compared with the other groups, and it is theoretically suitable for hemostasis.

### 3.3. In Vitro Hemostatic Ability of HPA4–6

**Figure 3:**
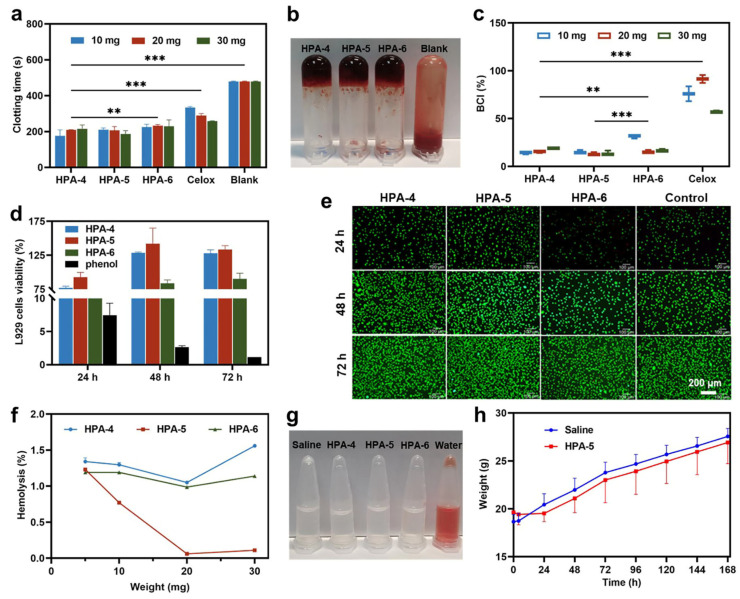
In vitro hemostatic properties and biocompatibility of HPA4–6 group. (**a**) In vitro clotting time plots for different levels of the HPA group, *n* = 3. (**b**) Photo of clotting time. (**c**) Blood clotting index values for different levels of the HPA group, *n* = 3, ** *p* < 0.01; *** *p* < 0.001. (**d**) Cytotoxicity in the HPA group for 24 h, 48 h, 72 h, *n* = 6 (**e**) Live/dead staining of cells in the HPA group. Scale bar: 200 μm. (**f**) The supernatant of each group of blood was measured with a microplate reader at 545 nm, *n* = 3. (**g**) The blood supernatant was collected after incubation with HPA, and hemolysis was observed after centrifugation. (**h**) Change in body weight of KM mice in the HPA-5 group, *n* = 5.

The results of the in vitro blood coagulation test at 10, 20, and 30 mg are shown in Figure 3a,b. Apparently, the clotting time of MBA_2_/KPS_m_ (HPA4–6) and Celox^TM^ was >30% faster compared with the blank group. The whole blood clotting time was significantly shorter with HPA4–6 (233 ± 6 s) than with the blank control (479 ± 2 s), and the hemostatic activity was superior to that of Celox^TM^, thus indicating better coagulation.

Subsequently, we performed an in vitro BCI test [43,47]. The more RBCs adsorbed by the material, the fewer that are ruptured by deionized water. Therefore, the better the coagulation effect of the hemostatic material, the lower the absorbance and BCI index. As shown in Figure 3c, the BCI indexes of HPA4–6 were significantly lower than that of Celox^TM^, indicating that HPA4–6 has better coagulation properties than Celox^TM^. Additionally, HPA-5 had the best clotting strength compared with the abovementioned three, which might be due to its higher water absorption and surface charge.

### 3.4. Biocompatibility of HPA-5

To further screen and evaluate the biocompatibility of HPA4–6, we performed hemolysis, cytotoxicity, and acute toxicity assays. The in vitro cytotoxicity of HPA4–6 was investigated using the MTT test. As shown in Figure 3d, the viability of L929 cells with other HPAs remained >75% over 3 days, except for HPA-6, which was cytotoxic after 24 h, maybe due to its low pH; this is consistent with the hemolysis rate observed in Figure 3g. A live/dead assay was used to observe the cell morphology (Figure 3e). L929 cells with a good morphology were stained green (live cells), and only a few cells were stained red (dead cells). In summary, HPA-4 and HPA-5 have excellent cell compatibility and can be used as hemostatic materials or wound dressings.

Furthermore, the hemolysis rate of each experimental group is shown in Figure 3f, with saline as the negative control group (0% hemolysis rate) and distilled water (100% hemolysis rate) as the positive control group. Compared with the positive control, the hemolysis rate in HPA-6 was <5%, in accordance with the range in ASTM-F756 [15]. HPA-5 had the lowest hemolysis rate (1.2%), indicating that HPA-5 had no significant damaging effect on RBCs and good hemocompatibility. The macroscopic colors of the supernatant after centrifugation in the HPA4–6 group, as well as the positive and negative controls after hemolysis, are shown in Figure 3g. HPA4–6 samples were almost colorless and transparent, similar to the negative controls, and the positive control was bright red. Hence, HPA-5 had optimal cytocompatibility and hemocompatibility.

To further demonstrate the safety of HPA-5, after an intraperitoneal HPA-5 injection, KM mice were generally in a good condition, and did not die, convulse, or prostrate. In particular, mouse activity decreased during the first 4 h after injection, and quickly returned to normal; this was due to the large amount of liquid administered at once, and they rapidly recovered after liquid absorption. The changes in body weight of KM mice over 7 days are shown in Figure 3h. The mice in the HPA-5 and saline groups presented with a steady increase in body weight, indicating the safety of HPA-5.

### 3.5. Adhesion Property of HPA-5

**Figure 4:**
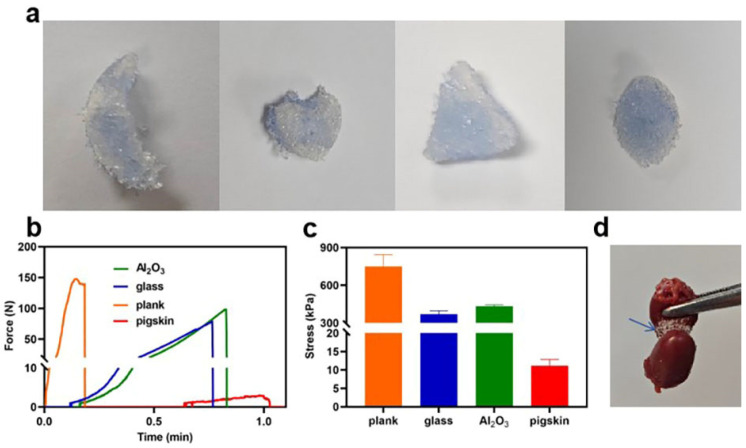
The adhesion property of HPA-5. (**a**) HPA-5 shape adaptability. (**b**) The force of HPA-5 on different matrix materials. (**c**) The adhesion strength of HPA-5 on different matrix materials, *n* = 3. (**d**) HPA effectively adhered to the rat hearts.

Hemostatic materials should exhibit appropriate shape adaptation and tissue adhesion to ensure that the injury site remains closed, and it should also form a physical barrier to avoid bacterial contamination [33]. The rapid water and blood absorption capacity of HPA allows it to be applied to irregular wounds (Figure 4a). The hydrogels tightly adhered to pigskin and various substrate surfaces, including glass, plank, and metal. The process for lap-shear strength measurement is illustrated in Figure 4b and the results are shown in Figure 4c. The corresponding adhesion strengths for plank, glass, metal, and pigskin were 749.02 ± 95.17, 371.63 ± 25.67, 433.54 ± 11.76, and 11.19 ± 1.64 kPa, respectively. Dried HPA granules do not adhere to dry physical surfaces, but they directly adhere to biological tissues such as wet pig skin. Therefore, the different states of HPA in the experiment resulted in a significant difference between the adhesion to the pigskin compared with the other samples. Additionally, HPA can closely adhere with biological tissues (Figure 4d) such as the heart, liver, spleen, lung, and kidney, likely due to hydrogen bonding or electrostatic interactions between the carboxyl groups in HPA and the amino groups in tissues. Therefore, the adhesion properties of HPA confer advantages for use as a wound dressing.

### 3.6. In Vivo Hemostatic Properties of HPA-5

**Figure 5:**
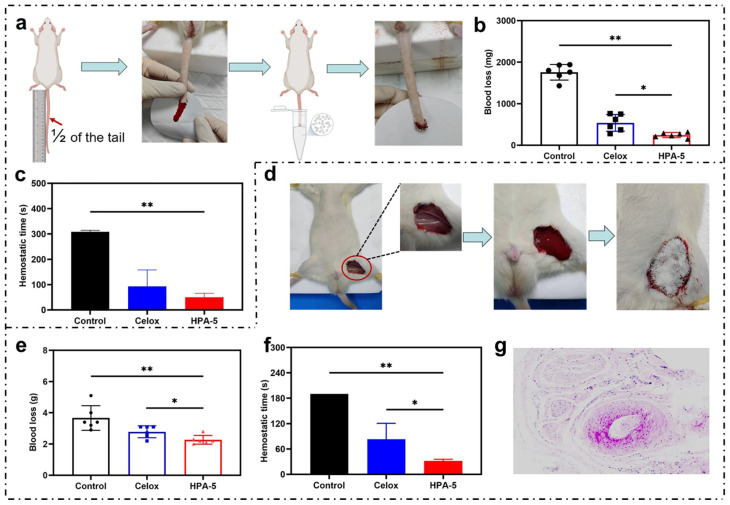
In vivo hemostatic properties of HPA-5. (**a**) Rat tail arteriovenous injury model, *n* = 6. (**b**) Blood loss in the rat tail arteriovenous injury experiment. (**c**) Hemostatic time in the rat tail arteriovenous injury experiment. (**d**) Rat femoral arteriovenous complete shear model, *n* = 6. (**e**) Blood loss in the rat femoral arteriovenous complete shear experiment. (**f**) Hemostatic time in the rat femoral arteriovenous complete shear experiment. * *p* < 0.05, ** *p* < 0.01. (**g**) Histological analysis of rat femoral arteriovenous complete shear model: artery (magnification, ×20).

The tail arteriovenous injury test in SD rats was performed to investigate the hemostatic effect of HPA in vivo. Celox^TM^ is one of the most successful commercial hemostatic wound dressings [46]. Compared with the blank group, the other test groups reduced the blood loss weight and shortened the bleeding time (Figure 5a–c). The blood loss with Celox^TM^ (538.3 ± 201.7 mg) was higher than with HPA. HPA exhibited the best hemostatic effect, with a blood loss of 245.0 ± 6.1 mg, which is about 86.1% lower than that found in the blank group. All treatment groups reduced the hemostasis time, exhibiting a similar trend to that of blood loss (Figure 5c). The hemostasis time in the blank was 308.3 ± 6.1 s, though HPA could have significantly reduced it to 50.0 ± 15.5 s, which is less than that of Celox^TM^ (93.3 ± 64.4 s). In terms of hemostasis time, HPA can be experimentally hemostatic within 50 s, whereas HPA composites require 80 s to achieve hemostasis [36], showing that the hemostatic activity of HPA alone is better.

The hemostatic performance of HPA-5 was evaluated using a total femoral arteriovenous complete shear model in rats (Figure 5d). The blood loss with HPA was only 2.3 ± 0.3 g lower than with Celox^TM^ (2.8 ± 0.4 g). In contrast to the average clotting time of the blank (190 ± 0 s), the clotting time of HPA was significantly shorter (31.7 ± 4.1 s). The hemostatic performance of HPA-5 was evaluated using two vessel injury bleeding models, showing a superior hemostatic performance in vivo compared with Celox^TM^. Histological analysis indicated that there was no inflammatory infiltration or pathological degeneration in the HPA group (Figure 5g). The above hemostatic performance evaluation indicated that HPA exhibited a better hemostatic performance than Celox^TM^, which may be due to the synergistic effect of HPA on the enrichment of RBCs and platelets, as well as the activation of coagulation factors.

### 3.7. Hemostatic Mechanism Based on Multiple Comprehensive Interactions of HPA-5

**Figure 6:**
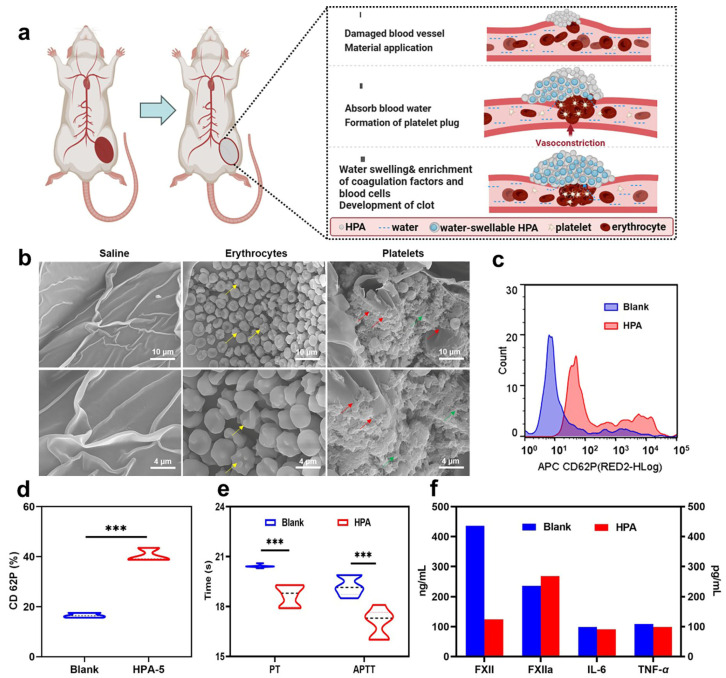
Hemostatic mechanism of HPA. (**a**) Coagulation mechanism of HPA. (**b**) SEM images of saline, erythrocyte, and platelet aggregation on the HPA: scale bar, scale bar, 10 μm for 2000×; scale bar, 5 μm for 5000×. Yellow arrows: erythrocytes were activated; red arrows: platelet activation; green arrows: formation of fibrin network. (**c**) FACS of platelet activation for the blank group and HPA group. (**d**) Platelet activation rate of the blank and HPA groups, *n* = 3. (**e**) PT and APTT activation time, *n* = 6. (**f**) HPA interactions with FXII, FXIIa, IL-6, and TNF-α, respectively. *** *p* < 0.001.

Due to the complexity of blood and coagulation processes, the hemostatic mechanism of most hemostatic materials is difficult to determine [48,49]. To investigate the hemostatic mechanism of HPA-5 (Figure 6a), we first explored the aggregation and activation of platelets and RBCs using hemostatic materials [50,51]. HPA-5 adsorbed a large quantity of RBCs and appeared tentacled, as shown in Figure 6b. Additionally, the complete morphology of RBCs further indicated that HPA-5 had good blood compatibility. Among HPA4–6, HPA-5 was the most effective regarding RBC aggregation (aggregation rate of 74.1 ± 0.1%) and fibrin network formation, having the shortest in vitro clotting time. When interacting with the HPA polymer, platelets were deformed (red arrows) and heavily aggregated in the activated state, as shown in Figure 6b. The platelet activation rate was used to indicate the hemostatic capacity of our material. Here, the PE-CD61 antibody marked platelets, and the APC-CD62P antibody labeled activated platelets [52]. FACS (fluorescence-activated cell sorting) showed a platelet activation rate of 16.54 ± 0.97% in the blank group and 40.46 ± 2.66% in the HPA-5 group (Figure 6c,d and Figure S4), further confirming the ability of HPA-5 to aggregate and activate platelets.

Thereafter, we performed a coagulation pathway activation assay. PT and APTT are two important indicators which determine the endogenous and exogenous pathways [53]. Compared with the blank group, the APTT and PT clotting times of the HPA-5 group significantly decreased by 11.35 ± 4.32% and 8.49 ± 3.06% (Figure 6e, Table S2). Accordingly, HPA-5 may promote the endogenous and exogenous pathways that activate the coagulation cascade reaction. TEG provides dynamic information on clot formation and stability by measuring the viscoelasticity of whole blood during coagulation, and it can evaluate the coagulation effect (Table S3) [24]. The R-value expresses the initial time of clot formation, and the K-value refers to the time from the end of the R time until the trace amplitude reaches 20 mm. The sum of the R and K values was the time of blood cell clot formation. The R and K values of HPA (R value was 1.07 ± 0.15 **, K value was 0.8 *) were significantly less than that of the blank control (R value was 2.50 ± 0.17, K value was 0.93 ± 0.06). This reduced R-value indicated that HPA lowers the coagulation initiation time, further demonstrating that HPA can activate the endogenous and exogenous pathways of the coagulation cascade reaction. Furthermore, the K-value indicated that HPA can accelerate the formation of blood clots, further demonstrating that HPA can activate platelets, accelerate the formation of a fibrin network, and facilitate blood clot formation.

Meanwhile, negatively charged hemostatic materials can rapidly activate endogenous coagulation pathways by activating FXII [45,46,54]. The quantitative analysis of HPA-5 interactions with FXII indicated a reduced content of the FXII factor in the HPA-5 group (123.77 ng/mL) compared with the blank group (435.25 ng/mL). Moreover, the content of the FXIIa factor in the HPA-5 group (269.00 ng/mL) was higher than in the blank group (236.45 ng/mL). As they were negatively charged on the surface of HPA particles, it was presumed that HPA activated coagulation factors XII to XIIa and triggered the endogenous coagulation pathway for hemostasis when they encountered blood. Hence, negative charges play a crucial role in activating the endogenous coagulation system (Figure 6f and Figure S5).

As HPA-5 has no group that adheres to RBCs, possible reasons for its excellent hemostatic effects are as follows. Firstly, it has high water absorption and can rapidly concentrate RBCs and platelets, thus promoting platelet aggregation and activation. Secondly, the three-dimensional mesh structure of HPA swells, providing a larger surface area for RBC and platelet adhesion after water absorption. Thirdly, the negative charge on the surface of the HPA can accelerate the activation of the clotting pathway, thus reducing the clotting time.

## 4. Conclusions

A polymeric HPA material was optimized by adjusting the relative contents of MBA and KPS. Among the nine HPAs produced at different ratios, HPA4–6 reacted completely with a dense pore structure, and it exhibited high water absorption. Additionally, HPA-4 and HPA-5 showed good biocompatibility and adhesion in vivo. Among them, HPA-5 exhibited rapid hemostatic activity in vitro and rapid hemostasis in a bleeding rat model, which can be applied to vessel-injured wounds. The good tissue adhesion ensured that the injury site remained closed, and it formed a physical barrier to avoid bacterial contamination. Furthermore, HPA can promote hemostasis by adhering to wounds, aggregating RBCs, activating platelets, accelerating coagulation pathways, and promoting fibrin crosslinking. This study provided the first systematic elucidation of the excellent hemostatic effects of HPA, indicating that HPA is a promising high-performance wound dressing biomaterial for traumatic hemorrhage.

## Figures and Tables

**Table 1:** Synthetic proportion of HPA.

	KPS_a_ (0.05%)	KPS_b_ (0.10%)	KPS_c_ (0.25%)
MBA_1_ (0.03%)	HPA-1	HPA-2	HPA-3
MBA_2_ (0.06%)	HPA-4	HPA-5	HPA-6
MBA_3_ (0.12%)	HPA-7	HPA-8	HPA-9

## Data Availability

Data are contained within the article.

## Supplementary Materials

The following supporting information can be downloaded at: https://www.mdpi.com/article/10.3390/polym16060863/s1. Figure S1: Energy Dispersive Spectrum of HPA-5: Red represents the C element, green represents the N element, purple represents the Na element, and blue represents the O element; Figure S2: Thermogravimetry (TG) of MBA_2_/KPS_m_ HPA group; Figure S3: HPA-5 effectively adhered to the rat tissues: (a) liver, (b) spleen, (c) lungs, (d) kidneys; Figure S4: Platelet activation results of HPA-5 using flow cytometry: (a) Blank group: the platelet activation rates were 15.64%, 16.41% and 17.57%. (b) HPA group: the platelet activation rates were 39.09%, 38.76% and 43.52%; Figure S5: Quantitative analysis of HPA-5 interactions with FXII, FXIIa, IL-6 and TNF-α; Table S1: Relative Elemental Content of HPA Energy Dispersive Spectrum; Table S2: In vitro activated partial thromboplastin time (APTT) and prothrombin time (PT) results of HPA-5. Data represent the mean ± SD (*n* = 6). *** *p* < 0.001 compared to Blank; Table S3: In vitro thromboelastogram (TEG) results of HPA-5. Data represent the mean ± SD (*n* = 3). * *p* < 0.05; ** *p* < 0.01 compared to Control.

## References

[1] Littlejohn L.F., Devlin J.J., Kircher S.S., Lueken R., Melia M.R., Johnson A.S. (2011). Comparison of Celox-A, ChitoFlex, WoundStat, and Combat Gauze Hemostatic Agents Versus Standard Gauze Dressing in Control of Hemorrhage in a Swine Model of Penetrating Trauma. Acad. Emerg. Med..

[2] Evans J.A., van Wessem K.J.P., McDougall D., Lee K.A., Lyons T., Balogh Z.J. (2010). Epidemiology of Traumatic Deaths: Comprehensive Population-Based Assessment. World J. Surg..

[3] Davis J.S., Satahoo S.S., Butler F.K., Dermer H., Naranjo D., Julien K., Van Haren R.M., Namias N., Blackbourne L.H., Schulman C.I. (2014). An Analysis of Prehospital Deaths: Who Can We Save?. J. Trauma Acute. Care.

[4] Hickman D.A., Pawlowski C.L., Sekhon U.D.S., Marks J., Gupta A.S. (2018). Biomaterials and Advanced Technologies for Hemostatic Management of Bleeding. Adv. Mater..

[5] Gao Y., Sarode A., Kokoroskos N., Ukidve A., Zhao Z., Guo S., Flaumenhaft R., Gupta A.S., Saillant N., Mitragotri S. (2020). A Polymer-Based Systemic Hemostatic Agent. Sci. Adv..

[6] Khoshmohabat H., Paydar S., Kazemi H.M., Dalfardi B. (2016). Overview of Agents Used for Emergency Hemostasis. Trauma Mon..

[7] Smith A.A., Ochoa J.E., Wong S., Beatty S., Elder J., Guidry C., McGrew P., McGinness C., Duchesne J., Schroll R. (2019). Prehospital Tourniquet Use in Penetrating Extremity Trauma: Decreased Blood Transfusions and Limb Complications. J. Trauma Acute Care Surg..

[8] Pedowitz R.A., Nordborg C., Rosenqvist A.-L., Rydevik B.L. (1991). Nerve Function and Structure Beneath and Distal to a Pneumatic Tourniquet Applied to Rabbit Hindlimbs. Scand. J. Plast. Recons..

[9] Déry R., Pelletier J., Jacques A., Clavet M., Houde J.J. (1965). Metabolic Changes Induced in the Limb during Tourniquet Ischaemla. Can. J. Anesth..

[10] Taboada G.M., Yang K., Pereira M.J.N., Liu S.S., Hu Y., Karp J.M., Artzi N., Lee Y. (2020). Overcoming the Translational Barriers of Tissue Adhesives. Nat. Rev. Mater..

[11] Hong C., Olsen B.D., Hammond P.T. (2022). A Review of Treatments for Non-Compressible Torso Hemorrhage (NCTH) and Internal Bleeding. Biomaterials.

[12] Shang X., Chen H., Castagnola V., Liu K., Boselli L., Petseva V., Yu L., Xiao L., He M., Wang F. (2021). Unusual Zymogen Activation Patterns in the Protein Corona of Ca-Zeolites. Nat. Catal..

[13] Liu W., Yang C., Gao R., Zhang C., Ou-Yang W., Feng Z., Zhang C., Pan X., Huang P., Kong D. (2021). Polymer Composite Sponges with Inherent Antibacterial, Hemostatic, Inflammation-Modulating and Proregenerative Performances for Methicillin-Resistant Staphylococcus Aureus-Infected Wound Healing. Adv. Health. Mater..

[14] Yuk H., Wu J., Sarrafian T.L., Mao X., Varela C.E., Roche E.T., Griffiths L.G., Nabzdyk C.S., Zhao X. (2021). Rapid and Coagulation-Independent Haemostatic Sealing by a Paste Inspired by Barnacle Glue. Nat. Biomed. Eng..

[15] Zhao X., Sun Y., Meng Z., Yang Z., Fan S., Ye T., Yang L., Li T., Gu R., Wu Z. (2022). Preparation and Characterization of Tranexamic Acid Modified Porous Starch and Its Application as a Hemostatic Agent. Int. J. Biol. Macromol..

[16] Bogdan Y., Helfet D.L. (2018). Use of Tourniquets in Limb Trauma Surgery. Orthop. Clin. N. Am..

[17] Beaman H.T., Shepherd E., Satalin J., Blair S., Ramcharran H., Serinelli S., Gitto L., Dong K.S., Fikhman D., Nieman G. (2022). Hemostatic Shape Memory Polymer Foams with Improved Survival in a Lethal Traumatic Hemorrhage Model. Acta. Biomater..

[18] Duggan M., Rago A., Sharma U., Zugates G., Freyman T., Busold R., Caulkins J., Pham Q., Chang Y., Mejaddam A. (2013). Self-Expanding Polyurethane Polymer Improves Survival in a Model of Noncompressible Massive Abdominal Hemorrhage. J. Trauma Acute Care.

[19] Huang A.P.-H., Lai D.-M., Hsu Y.-H., Tsai H.-H., Su C.-Y., Hsu S. (2021). An Anti-Inflammatory Gelatin Hemostatic Agent with Biodegradable Polyurethane Nanoparticles for Vulnerable Brain Tissue. Mat. Sci. Eng. C-Mater..

[20] Liang Y., Xu C., Liu F., Du S., Li G., Wang X. (2019). Eliminating Heat Injury of Zeolite in Hemostasis via Thermal Conductivity of Graphene Sponge. ACS Appl. Mater. Inter..

[21] Wang Y.W., Liu C.C., Cherng J.H., Lin C.S., Chang S.J., Hong Z.J., Liu C.C., Chiu Y.K., Hsu S.D., Chang H. (2019). Biological Effects of Chitosan-Based Dressing on Hemostasis Mechanism. Polymers.

[22] Hooshmand S., Mollazadeh S., Akrami N., Ghanad M., El-Fiqi A., Baino F., Nazarnezhad S., Kargozar S. (2021). Mesoporous Silica Nanoparticles and Mesoporous Bioactive Glasses for Wound Management: From Skin Regeneration to Cancer Therapy. Materials.

[23] Zhao Y., Li J., Leng F., Lv S., Huang W., Sun W., Jiang X. (2020). Degradable Porous Carboxymethyl Chitin Hemostatic Microspheres. J. Biomat. Sci.-Polym. Ed..

[24] Liu T., Zhang Z., Liu J., Dong P., Tian F., Li F., Meng X. (2022). Electrospun Kaolin-Loaded Chitosan/PEO Nanofibers for Rapid Hemostasis and Accelerated Wound Healing. Int. J. Biol. Macromol..

[25] Yu L., Shang X., Chen H., Xiao L., Zhu Y., Fan J. (2019). A Tightly-Bonded and Flexible Mesoporous Zeolite-Cotton Hybrid Hemostat. Nat. Commun..

[26] Slezak P., Keibl C., Redl H., Labahn D., Gulle H. (2020). An Efficacy Comparison of Two Hemostatic Agents in a Porcine Liver Bleeding Model: Gelatin/Thrombin Flowable Matrix versus Collagen/Thrombin Powder. J. Investig. Surg..

[27] Cao X., Zhang Z., Sun L., Luo Z., Zhao Y. (2022). Multifunctional Fish Gelatin Hydrogel Inverse Opal Films for Wound Healing. J. Nanobiotechnol..

[28] Pandey S., Son N., Kang M. (2022). Synergistic Sorption Performance of Karaya Gum Crosslink Poly(Acrylamide-Co-Acrylonitrile) @ Metal Nanoparticle for Organic Pollutants. Int. J. Biol. Macromol..

[29] Tie L., Ke Y., Gong Y., Zhang W., Deng Z. (2022). Nanocellulose Fine-Tuned Poly(Acrylic Acid) Hydrogel for Enhanced Diclofenac Removal. Int. J. Biol. Macromol..

[30] Kim J., Krishna-Subbaiah N., Wu Y., Ko J., Shiva A., Sitti M. (2023). Enhanced Flexible Mold Lifetime for Roll-to-Roll Scaled-Up Manufacturing of Adhesive Complex Microstructures. Adv. Mater..

[31] Song Z., Liu X., Ding J., Liu J., Han X., Deng Y., Zhong C., Hu W. (2022). Poly(Acrylic Acid)-Based Composite Gel Polymer Electrolytes with High Mechanical Strength and Ionic Conductivity toward Flexible Zinc–Air Batteries with Long Cycling Lifetime. ACS Appl. Mater. Inter..

[32] Xu H.-G., Liang Q.-L., Li L., Qi G.-F., Wang L., Zhan L.-N., Ding M.-R., Zhang K., Cui X. (2022). Biomimetic Peptide Nanoparticles Participate in Natural Coagulation for Hemostasis and Wound Healing. Biomater. Sci..

[33] Zhang M., Yang Q., Hu T., Tang L., Ni Y., Chen L., Wu H., Huang L., Ding C. (2022). Adhesive, Antibacterial, Conductive, Anti-UV, Self-Healing, and Tough Collagen-Based Hydrogels from a Pyrogallol-Ag Self-Catalysis System. ACS Appl. Mater. Inter..

[34] Ma C., Zhao J., Zhu C., Jiang M., Ma P., Mi Y., Fan D. (2022). Oxidized Dextran Crosslinked Polysaccharide/Protein/Polydopamine Composite Cryogels with Multiple Hemostatic Efficacies for Noncompressible Hemorrhage and Wound Healing. Int. J. Biol. Macromol..

[35] Yu X., Gao Z., Mu J., Lian H., Meng Z. (2023). Gelatin/Calcium Chloride Electrospun Nanofibers for Rapid Hemostasis. Biomater. Sci..

[36] Xiang J., Wang Y., Yang L., Zhang X., Hong Y., Shen L. (2022). A Novel Hydrogel Based on Bletilla Striata Polysaccharide for Rapid Hemostasis: Synthesis, Characterization and Evaluation. Int. J. Biol. Macromol..

[37] Yu J., Qin Y., Yang Y., Zhao X., Zhang Z., Zhang Q., Su Y., Zhang Y., Cheng Y. (2023). Robust Hydrogel Adhesives for Emergency Rescue and Gastric Perforation Repair. Bioact. Mater..

[38] Zhu H., Xu G., He Y., Mao H., Kong D., Luo K., Tang W., Liu R., Gu Z. (2022). A Dual-Bioinspired Tissue Adhesive Based on Peptide Dendrimer with Fast and Strong Wet Adhesion. Adv. Health. Mater..

[39] Fonseca R.G., De Bon F., Pereira P., Carvalho F.M., Freitas M., Tavakoli M., Serra A.C., Fonseca A.C., Coelho J.F.J. (2022). Photo-Degradable, Tough and Highly Stretchable Hydrogels. Mater. Today Bio..

[40] Matthay Z.A., Fields A.T., Nunez-Garcia B., Park J.J., Jones C., Leligdowicz A., Hendrickson C.M., Callcut R.A., Matthay M.A., Kornblith L.Z. (2022). Importance of Catecholamine Signaling in the Development of Platelet Exhaustion after Traumatic Injury. J. Thromb. Haemost..

[41] Jungsinyatam P., Suwanakood P., Saengsuwan S. (2022). Multicomponent Biodegradable Hydrogels Based on Natural Biopolymers as Environmentally Coating Membrane for Slow-Release Fertilizers: Effect of Crosslinker Type. Sci. Total Environ..

[42] Roig-Sanchez S., Kam D., Malandain N., Sachyani-Keneth E., Shoseyov O., Magdassi S., Laromaine A., Roig A. (2022). One-Step Double Network Hydrogels of Photocurable Monomers and Bacterial Cellulose Fibers. Carbohyd. Polym..

[43] Abdullah T., Colombani T., Alade T., Bencherif S.A., Memić A. (2021). Injectable Lignin-Co-Gelatin Cryogels with Antioxidant and Antibacterial Properties for Biomedical Applications. Biomacromolecules.

[44] Feng C., Li J., Wu G.S., Mu Y.Z., Kong M., Jiang C.Q., Cheng X.J., Liu Y., Chen X.G. (2016). Chitosan-Coated Diatom Silica as Hemostatic Agent for Hemorrhage Control. ACS Appl. Mater. Inter..

[45] Zheng C., Bai Q., Wu W., Han K., Zeng Q., Dong K., Zhang Y., Lu T. (2021). Study on Hemostatic Effect and Mechanism of Starch-Based Nano-Microporous Particles. Int. J. Biol. Macromol..

[46] Zheng C., Zeng Q., Pimpi S., Wu W., Han K., Dong K., Lu T. (2020). Research Status and Development Potential of Composite Hemostatic Materials. J. Mater. Chem. B.

[47] Zhang Z., Kuang G., Zong S., Liu S., Xiao H., Chen X., Zhou D., Huang Y. (2018). Sandwich-Like Fibers/Sponge Composite Combining Chemotherapy and Hemostasis for Efficient Postoperative Prevention of Tumor Recurrence and Metastasis. Adv. Mater..

[48] Huang Y., Fan C., Liu Y., Yang L., Hu W., Liu S., Wang T., Shu Z., Li B., Xing M. (2022). Nature-Derived Okra Gel as Strong Hemostatic Bioadhesive in Human Blood, Liver, and Heart Trauma of Rabbits and Dogs. Adv. Healthc. Mater..

[49] Wang Y., Zhao Y., Qiao L., Zou F., Xie Y., Zheng Y., Chao Y., Yang Y., He W., Yang S. (2021). Cellulose Fibers-Reinforced Self-Expanding Porous Composite with Multiple Hemostatic Efficacy and Shape Adaptability for Uncontrollable Massive Hemorrhage Treatment. Bioact. Mater..

[50] Peng X., Xu X., Deng Y., Xie X., Xu L., Xu X., Yuan W., Yang B., Yang X., Xia X. (2021). Ultrafast Self-Gelling and Wet Adhesive Powder for Acute Hemostasis and Wound Healing. Adv. Funct. Mater..

[51] Du X., Wu L., Yan H., Jiang Z., Li S., Li W., Bai Y., Wang H., Cheng Z., Kong D. (2021). Microchannelled Alkylated Chitosan Sponge to Treat Noncompressible Hemorrhages and Facilitate Wound Healing. Nat. Commun..

[52] Zhao X., Liang Y., Guo B., Yin Z., Zhu D., Han Y. (2021). Injectable Dry Cryogels with Excellent Blood-Sucking Expansion and Blood Clotting to Cease Hemorrhage for Lethal Deep-Wounds, Coagulopathy and Tissue Regeneration. Chem. Eng. J..

[53] Liu C., Liu X., Liu C., Wang N., Chen H., Yao W., Sun G., Song Q., Qiao W. (2019). A Highly Efficient, in Situ Wet-Adhesive Dextran Derivative Sponge for Rapid Hemostasis. Biomaterials.

[54] Sparkenbaugh E.M., Henderson M.W., Miller-Awe M., Abrams C., Ilich A., Trebak F., Ramadas N., Vital S., Bohinc D., Bane K.L. (2023). Factor XII Contributes to Thrombotic Complications and Vaso-Occlusion in Sickle Cell Disease. Blood.

