# Supplementary material for: Highly Efficient Hemostatic Cross-Linked Polyacrylate Polymer Dressings for Immediate Hemostasis
Source: Polymers (Basel). 2024 Mar 21;16(6):863. doi: 10.3390/polym16060863 (PMC10974503; doi:10.3390/polym16060863)
Supplement: Supplementary file 1 [file polymers-16-00863-s001.zip › polymers-2885143-supplementary.pdf]

polymers

Supplementary

Highly Efficient Hemostatic Cross-Linked Polyacrylate Polymer Dressings for Immediate Hemostasis

Tong Ye, Zhiyuan Yang, Ruolin Hao, Jinnan Guo, Guifang Dou, Zhiyun Meng, Shuchen Liu, Ruolan Gu, Zhuona Wu, Yunbo Sun, Peng Han, Yiguang Jin * and Hui Gan *

Department of Pharmaceutical Sciences, Beijing Institute of Radiation Medicine, Beijing 100850, China
* Correspondence: jinyg@sina.com (Y.J.); ganh2003@163.com (H.G.)

Figure S1. Energy Dispersive Spectrum of HPA-5: Red represents the C element, green represents the N element, purple represents the Na element, and blue represents the O element.
Figure S2. Thermogravimetry (TG) of MBA_2/KPS_m HPA group.
Figure S3. HPA-5 effectively adhered to the rat tissues: (a) liver, (b) spleen, (c) lungs, (d) kidneys.
Figure S4. Platelet activation results of HPA-5 using flow cytometry: (a) Blank group: the platelet activation rates were 15.64%, 16.41% and 17.57%. (b)HPA group: the platelet activation rates were 39.09%, 38.76% and 43.52%.
Figure S5. Quantitative analysis of HPA-5 interactions with FXII, FXIIa, IL-6 and TNF-α.

Table S1. Relative Elemental Content of HPA Energy Dispersive Spectrum.
Table S2. *In vitro* activated partial thromboplastin time (APTT) and prothrombin time (PT) results of HPA-5. Data represent the mean ± SD (n = 6). ***$p < 0.001$ compared to Blank.
Table S3. *In vitro* thromboelastogram (TEG) results of HPA-5. Data represent the mean ± SD (n = 3). *$p < 0.05$; **$p < 0.01$ compared to Control.

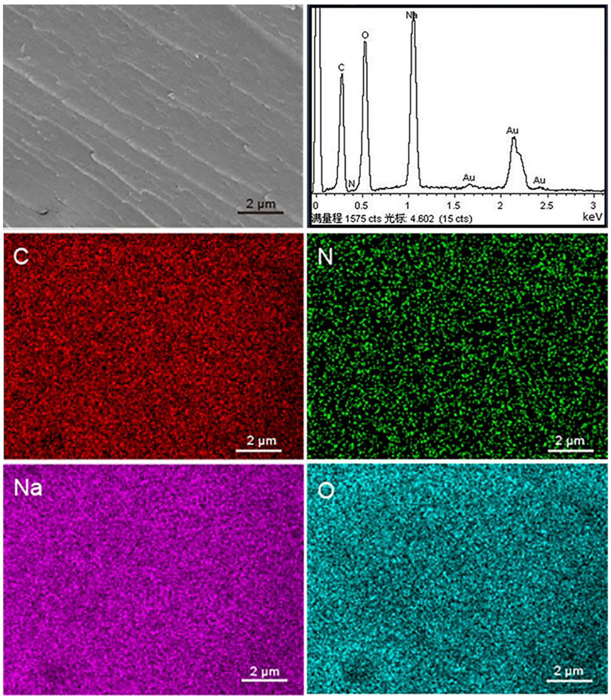

Figure S1. Energy Dispersive Spectrum of HPA-5: Red represents the C element, green represents the N element, purple represents the Na element, and blue represents the O element.

Table S1. Relative Elemental Content of HPA Energy Dispersive Spectrum

HPA	C	O	Na	N	N/C
**1**	48.3	30.4	21.2	0.1	0.00207
**2**	44.63	42.7	11.86	0.81	0.018149
**3**	45.1	28.9	25.7	0.3	0.006652
**4**	42.16	44.9	11.65	1.29	0.030598
**5**	40.42	45.85	11.92	1.82	0.045027
**6**	40.79	43.61	14.04	1.56	0.038245
**7**	42.1	28.7	27.3	1.9	0.045131
**8**	42.27	39.18	15.68	2.86	0.06766
**9**	41.79	42.76	13.23	2.22	0.053123

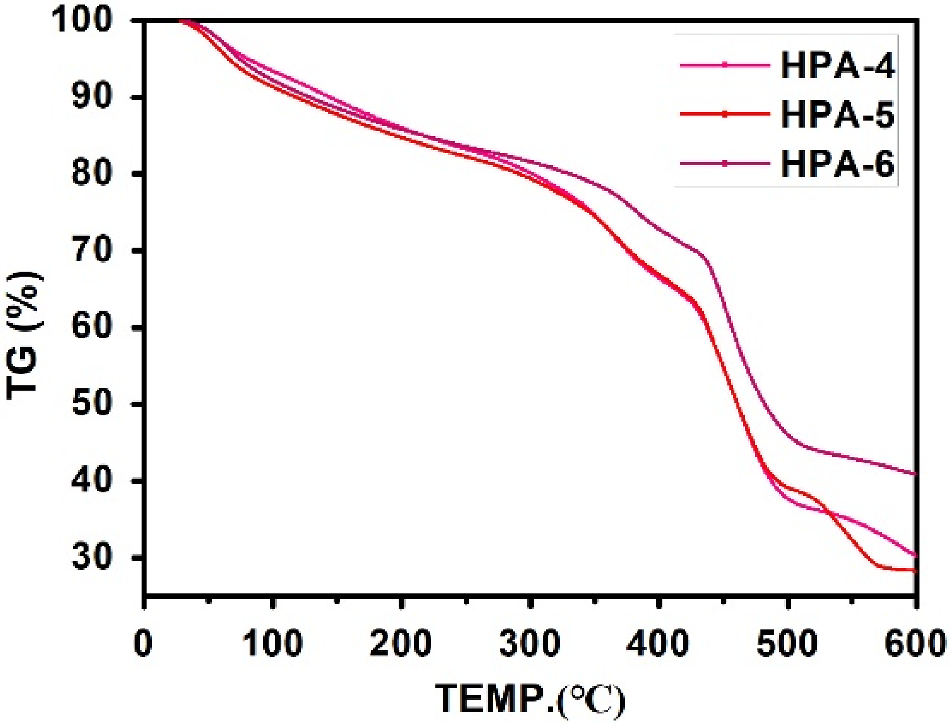

Figure S2. Thermogravimetry (TG) of MBA_2/KPS_m HPA group.

The thermal stability of HPA as shown in Figure S2. MBA_2/KPS_m of HPA lost about 8% weight below 100°C for free water evaporation. The weight loss was about 12% from 340°C to 430°C, presumably due to the decomposition of a few linear oligomers in the sample. The rapid weight loss of 23% at 430°C~500°C was caused by the decomposition of the macromolecular chain of the cross-linked HPA in the sample, indicating that HPA has the property of high temperature resistance and is suitable for stable storage as biomedical material.

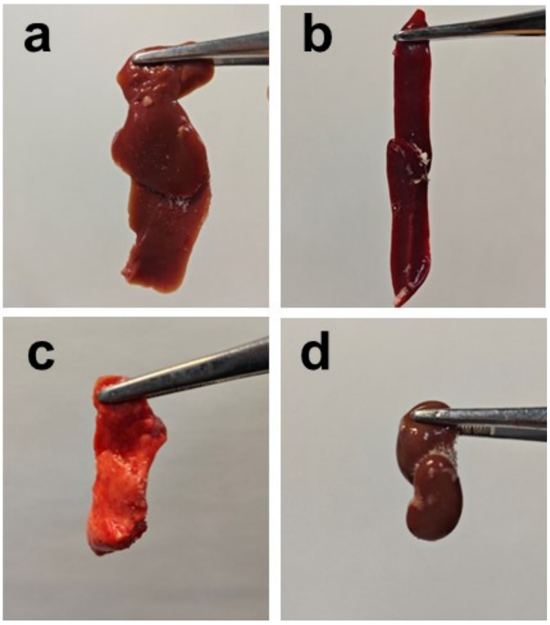

Figure S3. HPA-5 effectively adhered to the rat tissues: (a) liver, (b) spleen, (c) lungs, (d) kidneys.

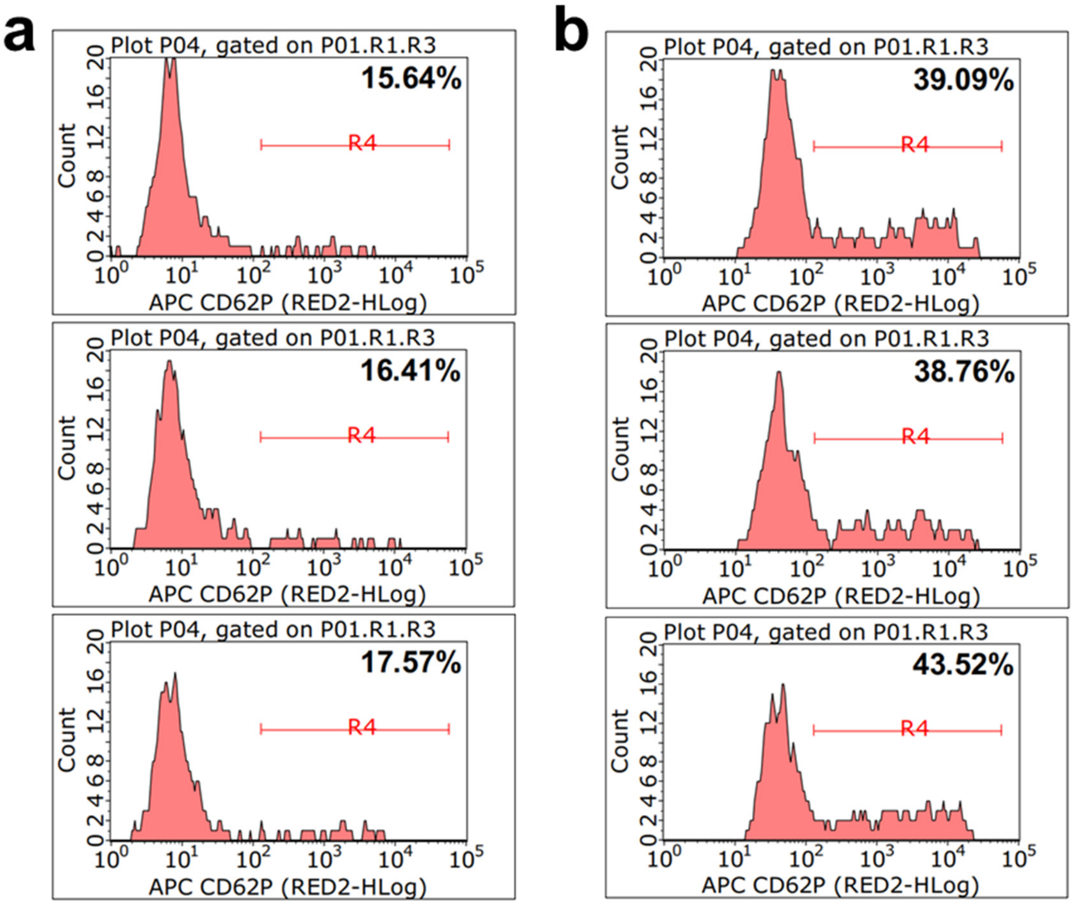

Figure S4. Platelet activation results of HPA-5 using flow cytometry: (a) Blank group the platelet activation rates were 15.64%, 16.41% and 17.57%. (b) HPA group: the platelet activation rates were 39.09%, 38.76% and 43.52%.

Platelets were activated to produce tentacles that contributed to the formation of a more stable blood clot, hence the platelet activation rate showed the hemostatic capacity of the material to a certain extent. APC-CD62P and PE-CD61 are monoclonal antibodies against rat-derived CD62P for flow cytometric analysis, which are used for *in vitro* detection of the expression of the platelet marker CD62P in rat blood. Theoretically, the PE-CD61 antibody will label all platelets, and the APC-CD62P antibody will label activated platelets. As shown in Fig S4, the platelet activation rate was 16.54±0.97% in the blank group and 40.46±2.66% in the HPA group. HPA had the ability to activate platelets compared to the blank group, probably due to the potent blood-sucking ability of HPA with high water-absorbency. At the same time, HPA was exposed to water with Na^+ leaving the polymer ionic chain. Mutual repulsive forces generated by a large number of negatively charged polymer ions caused the polymer mesh bundles to gradually stretch from the entangled state, providing a larger deposition surface for platelets. Thus, more platelets were attracted and concentrated, increasing the activation rate of platelets.

Table S2. *In vitro* activated partial thromboplastin time (APTT) and prothrombin time (PT) results of HPA-5. Data represent the mean ± SD (n = 6). ***$p < 0.001$ compared to Blank.

Samples	PT (s)		APTT (s)	
	Blank	HPA-5	Blank	HPA-5
1	20.4	19.3	18.5	16.0
2	20.4	19.0	18.8	16.1
3	20.6	18.0	19.1	17.4
4	20.4	19.3	19.2	17.2
5	20.3	18.6	19.9	17.5
6	20.4	17.9	19.9	18.1
mean ± SD	20.4 ± 0.1	18.7 ± 0.6***	19.2 ± 0.6	17.1 ± 0.8***

Table S3. *In vitro* thromboelastogram (TEG) results of HPA-5. Data represent the mean ± SD (n = 3). *$p < 0.05$; **$p < 0.01$ compared to Control.

Samples	R (min)	K (min)	Angle (deg)	MA (mm)
Blank	2.50 ± 0.17	0.93 ± 0.06	72.90 ± 5.39	63.67 ± 4.56
HPA	1.07 ± 0.15**	0.80 ± 0.00*	83.73 ± 1.25*	66.90 ± 1.61

Activation of coagulation factors is the trigger mechanism for blood coagulation. According to the different forms of triggering coagulation process, the blood coagulation pathway is divided into endogenous and extrinsic coagulation, which were evaluated by measuring activated partial thromboplastin time (APTT) and prothrombin time (PT) respectively. APTT is associated with endogenous pathway coagulation factors such as factors XII, XI, VIII, and IX, reflecting the function of the endogenous coagulation system. PT is related to exogenous pathway coagulation factors such as tissue factor and coagulation factor VII, to reflect the function of exogenous coagulation system. Hence, APTT and PT were measured to clarify the hemostatic mechanism of HPA. As shown in Table S2, the APTT values of the blank group and HPA group were significantly different ($p < 0.001$), which indicated that HPA played a great role in triggering the intrinsic coagulation pathway. This may be due to the activation of FXII by negative charges on the surface and water-absorption of HPA. The PT values of HPA was statistically lower than the blank group ($p < 0.001$), which could be caused by the HPA absorbing water and concentrating the tissue factors. All the results indicated that HPA could promote blood coagulation through both endogenous and exogenous pathways.

The evaluation of coagulation function can be analyzed from the dynamic coagulation process of whole blood, namely thromboelastography (TEG). It provides dynamic information such as clot formation and stability by measuring the viscoelastic properties of whole blood during coagulation, reflecting the whole process of hemostasis in vivo. There are several key evaluation parameters in this process. The R value represents the period of latency between the start of detection and the formation of the first fibrin clot, which is the time for sequential activation of coagulation factors. As shown in Table S3, the blood of HPA group started coagulation from 1.07 ± 0.15 min (R), which was statistically faster than blank (2.50 ± 0.17 min, $p <$

0.01). The K value refers to the time from the end of the R time until the trace amplitude reaches 20 mm. The summation of R and K value is the time of blood cell clot formation. Compared with the blank group, the sum of R and K values for each HPA group sample significantly shortened. Angle (α) refers to the angle between the tangent line and the horizontal line from the point of blood clot formation to the maximum curve arc of the trace, which reflects the coagulation of the rat. MA reflects the maximum strength or firmness of the developing clot and the stability of the clot formation. As shown in Table S3, the Angle and MA of HPA group increased to different degrees compared with those of the blank group, which proved that HPA could be beneficial to the formation and stability of blood clots. When HPA was in initial contact with blood, the smaller R and K values of TEG indicated that HPA had the ability to shorten the activation time of coagulation factors, which was consistent with the results of PT and APTT.

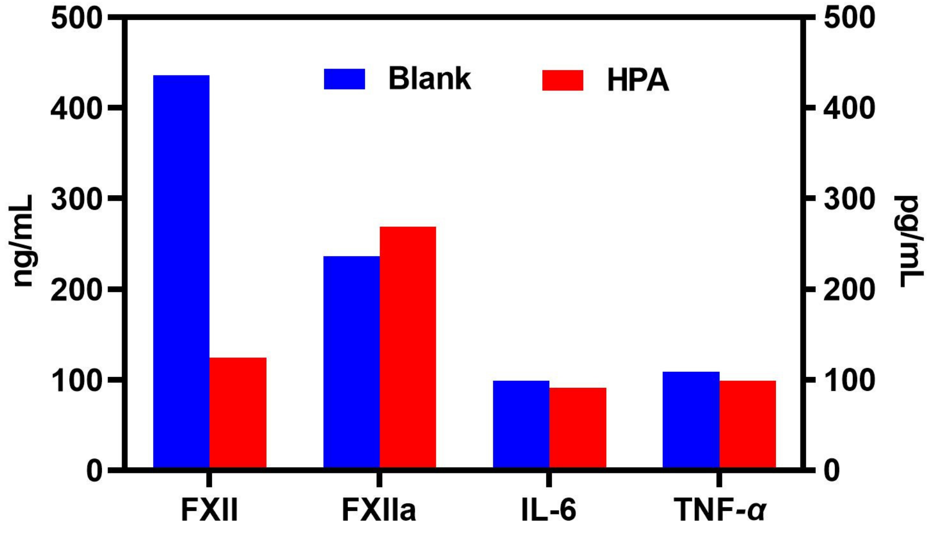

Figure S5. Quantitative analysis of HPA-5 interactions with FXII, FXIIa, IL-6 and TNF-α.

It is known that negatively charged particles on the surface activate coagulation factors XII to XIIa, which trigger the endogenous coagulation pathway for hemostasis. After HPA interacted with factor FXII in whole blood plasma, the plasma level of FXII significantly decreased from 435.69 ng/mL to 124.59 ng/mL and the level of FXIIa increased from 236.45 ng/mL to 269.00 ng/mL. To exclude the adsorption of HPA itself to protein factors in plasma, the interactions of IL-6 and TNF-α factors with HPA were analyzed. The IL-6 factor level was reduced from 98.42 pg/mL to 90.72 pg/mL, and the TNF-α factor level was reduced from 108.84 pg/mL to 98.84 pg/mL, which indicated that HPA specifically reacted with FXII and FXIIa in the blood, rather than a non-specific adsorption. The results of the interaction of HPA with four factors provided further evidence that HPA accelerated the activation of the endogenous coagulation pathway.
